# Hitchhiking or hang gliding? Dispersal strategies of two cereal-feeding eriophyoid mite species

**DOI:** 10.1007/s10493-021-00661-z

**Published:** 2021-10-05

**Authors:** Agnieszka Majer, Alicja Laska, Gary Hein, Lechosław Kuczyński, Anna Skoracka

**Affiliations:** 1 Population Ecology Lab, Faculty of Biology, Adam Mickiewicz University in Poznań, Poznań, Poland; 2 Department of Entomology, University of Nebraska-Lincoln, Lincoln, USA

**Keywords:** *Abacarus hystrix*, *Aceria tosichella*, Aerial dispersal, Cereal rust mite, Eriophyidae, Phoresy, Wheat curl mite

## Abstract

Dispersal shapes the dynamics of populations, their genetic structure and species distribution; therefore, knowledge of an organisms’ dispersal abilities is crucial, especially in economically important and invasive species. In this study, we investigated dispersal strategies of two phytophagous eriophyoid mite species: *Aceria tosichella* (wheat curl mite, WCM) and *Abacarus hystrix* (cereal rust mite, CRM). Both species are obligatory plant parasites that infest cereals and are of economic significance. We investigated their dispersal success using different dispersal agents: wind and vectors. We hypothesised that in both mite species the main mode of dispersal is moving via wind, whereas phoretic dispersal is rather accidental, as the majority of eriophyoid mite species do not possess clear morphological or behavioural adaptations for phoresy. Results confirmed our predictions that both species dispersed mainly with wind currents. Additionally, WCM was found to have a higher dispersal success than CRM. Thus, this study contributes to our understanding of the high invasive potential of WCM.

## Introduction

Dispersal is an important biological process that determines the dynamics and spatial distribution of populations, shapes their genetic structure, and affects evolutionary processes, such as local adaptation and speciation (Ronce 2007; Clobert et al. 2012). A high dispersal ability can lead to accelerated spread in the field and hence can influence the range expansion of organisms by potentially increasing invasion into a new habitat (Dunning et al. 1995; Hanski and Gilpin 1997; Hanski 1999; Clobert et al. 2001; Kot and Lewis 1996; Phillips et al. 2008; Travis et al. 2009). Thus, knowledge of the dispersal ability of pests or other organisms of economic significance, like eriophyoid mites, is of crucial importance to develop effective monitoring and management tactics.

Eriophyoid mites (Acariformes: Eriophyoidea) are obligatory plant parasites, some inducing a great adverse impact on agriculture and forestry due to a high invasive potential (Navia et al. 2010). Thus, there is a huge demand for empirical data on their dispersal abilities that would enable predictions of future expansion of harmful species. There is some evidence that eriophyoid mites can disperse actively for short distances, i.e., by walking within or between plants (Michalska et al. 2010; Galvão et al. 2012; Melo et al. 2014; Majer et al. 2021a). However, this mode of dispersal is extremely limited due to their minute size (length ca. 0.2 mm) and lack of adaptations that facilitate efficient active dispersal (Lindquist and Oldfield 1996). Eriophyoid mites are known to increase their ranges by aerial dispersal (Lindquist and Oldfield 1996; Sabelis and Bruin 1996; Zhao and Amrine 1997a, b; Thomas and Hein 2003). However, phoretic dispersal with invertebrate and vertebrate vectors has also been hypothesised to be a mode of eriophyoid mite transfer (Massee 1928; Gibson and Painter 1957; Shvanderov 1975; Waite and McAlpine 1992; Jeppson et al. 1975; Schliesske 1990; Liu et al. 2016), although experimental studies on phoretic dispersal are much scarcer than those on aerial dispersal (Michalska et al. 2010). Generally, the dispersal strategies in the majority of eriophyoid mite species remain untested (Michalska et al. 2010). It is important to note that aerial and phoretic dispersal are commonly considered passive forms of dispersal because the organisms cannot control their movement (Clobert et al. 2012; but see Washburn and Washburn 1984; Jung and Croft 2001). However, during the departure (emigration) phase, organisms often undertake behaviours that increase the probability of their dispersal (Bell et al. 2005; Reynolds et al. 2007; Osakabe et al. 2008; Reynolds and Reynolds 2009). Thus, passive dispersal may include an active component.

In this study, various modes of passive dispersal were investigated for grass-feeding eriophyoid species: wheat curl mite (WCM) *Aceria tosichella* Keifer and cereal rust mite (CRM) *Abacarus hystrix* (Nalepa). Passive dispersal is defined as movement driven primarily by external forces where there is no control over the direction or distance travelled. Both species infest different grass hosts, including cereals, and are vectors of plant viruses (Oldfield and Proeseler 1996; Mahmood et al. 1998; Byamukama et al. 2012; Navia et al. 2013; Oliveira-Hofman et al. 2015). WCM is one of the most important pests of common wheat (*Triticum aestivum* L.) (Navia et al. 2010, 2013), which is a crucial food grain source for humans (Shewry and Hey 2015). The mechanism of aerial dispersal of WCM has been tested several times in the field (Slykhuis 1953, 1955; Pady 1955; Staples and Allington 1956, 1959; Nault and Styer 1969; Harvey and Martin 1980, 1988; Harvey et al. 1990; Brey 1998; Liu et al. 2005; Umina et al. 2016) and experimentally (e.g. Thomas and Hein 2003; Kiedrowicz et al. 2017; Laska et al. 2019; Overmyer 2020; Kuczyński et al. 2020), whereas studies on the ability of CRM to effectively spread by wind are much scarcer (Nault and Stryer 1969; Kuczyński et al. 2020). Although the role of phoresy in the spread of WCM has been discussed in literature for more than 50 years (Slykhuis 1955; Michalska et al. 2010; Kiedrowicz et al. 2017), critical empirical investigations on the possibility of dispersal with vectors in those two mite species are scarce. There are only two published experimental tests of the ability of WCM to disperse with insect vectors, i.e., aphids (Gibson and Painter 1957) or an artificial vertebrate vector (Kuczyński et al. 2020). However, there has been no experimental study testing the possibility of phoretic dispersal in CRM.

Here, we fill this gap and assess the dispersal success of WCM and CRM using wind and phoretic vectors. We investigated aerial dispersal in wind tunnels (Majer et al. 2021a). To test phoretic dispersal, we used *Anaphothrips obscurus* Müller (Insecta: Thysanoptera) and a robot constructed from Lego Mindstorm kits (a device built according to Kuczyński et al. 2020), as potential small and large vectors, respectively. We hypothesised that wind is the most successful mode of dispersal of both eriophyoid mites, whereas phoretic dispersal is rather accidental, due to the lack of clear morphological (Lindquist and Oldfield 1996) or behavioural (Kiedrowicz et al. 2017) adaptations to phoresy in both mite species.

## Materials and methods

### Study system

Stock colonies of WCM and CRM were maintained on bread wheat (var. Muszelka) growing in pots and kept in rearing cages consisting of metal frames wrapped with nylon bags to protect against contamination, at room conditions (22–24 °C, L12:D12 photoperiod, 45% RH). WCM specimens originated from common wheat *Triticum aestivum* in Choryń, Poland (52°02′36″ N, 16°46′02″ E), and CRM specimens originated from quack grass, *Elymus repens* (L.) Gould, in Poznań, Poland (52°28′04″ N, 16°55′36″ E). WCM and CRM are species complexes consisting of several distinct genetic lineages (Skoracka and Dabert 2010; Skoracka et al. 2012, 2018a; Szydło et al. 2015; Laska et al. 2018). The stock colonies were established from WCM lineage MT-1 (also called Type 2; Hein et al. 2012; Skoracka et al. 2018b) (GenBank acc. no: JF920077) (Skoracka et al. 2012) and CRM *Abacarus hystrix* complex 2 (GenBank acc. no: FJ387550.1) (Skoracka and Dabert 2010; Laska et al. 2018) based on molecular identification using a cytochrome c oxidase subunit I (COI) gene fragment (Dabert et al. 2010; Skoracka and Dabert 2010). Hereafter, for simplicity, we use WCM for WCM MT-1 and CRM for CRM complex 2. When referring to the species complexes in general (not a particular lineage), we call it WCM sensu lato or CRM sensu lato*.*

### Experimental design

Three factors potentially playing a role in passive dispersal were tested (wind and two types of phoretic vectors) along with a control with no dispersal factor. Tests of dispersal were performed in transparent tunnels constructed according to Kuczyński et al. (2020) with modifications, as described below. The tunnels were made of plexiglass (PMMA) tubes (11 cm diameter, 50 cm long). The 50-cm long tunnels were used on the basis of previous studies (e.g., Kuczyński et al. 2020; Overmyer 2020) and pilot tests. Tests showed that a 50-cm distance allows for aerial mite dispersal and the active movement of insect vectors, but not the active movement (walking) of mites. In this way, dispersal via vectors and wind could be tested, and the possibility of mites dispersing to target plants by walking was excluded. Wheat plants (ca. 18 cm high) infested with WCM or CRM were cut from the stock colonies, and mite specimens were counted under a stereomicroscope (Olympus SZ40). These cut wheat shoots infested with mites were next used in experiments as the source shoots, and each one served as a single experimental unit. Mean density of mites per shoot for each variant including control was 1205.6 (range 290–2500, *n* = 43) for WCM and 1476.3 (range 400–2500, *n* = 40) for CRM. Source shoots were installed at one end of the tunnel by using clips. At the other end of the tunnel, we placed pots (9 cm diameter) containing 10 uninfested wheat plants (up to 10 days old, ca. 10 cm high, hereafter called ‘target plants’). In all tests, the distances between the source shoot and target plants were 50 ± 2 cm and the exposure period was 48 h. After 48-h dispersal sessions, source shoots were inspected under a stereomicroscope to assess the condition of plants and to determine the presence of live mites (Table 1). All exposure tests started in the afternoon (about 2:00–3:00 pm) and were performed at room temperature (22 °C). Technical adaptations and procedures depending on the tested dispersal mode were performed as described below.

**Table 1:** Mean number of individuals on the source shoot at the beginning of the experiment (*N*), mean percentage of *Aceria tosichella* (wheat curl mite, WCM) and *Abacarus hystrix* (cereal rust mite, CRM) mites (i.e., residents) remaining alive on source shoots after 48 h of exposure to dispersal agent, and mean number of colonists (*C*) after a 14-day incubation period (in parentheses 95% confidence intervals)

Species	Dispersal agent	No. mites/source shoot (*N*)	% Residents/source shoot	No. colonists/target shoots (*C*)
WCM	Wind	1290.0 (991.0–1643.8)	0.30 (0.21–0.40)	207.1 (198.31–216.15)
	Insect vector	1079.0 (807.8–1405.0)	4.27 (3.90–4.67)	0
	Robot	1595.0 (1259.6–1985.2)	0.62 (0.51–0.75)	0
CRM	Wind	1585.0 (1250.7–1974.1)	0	93.3 (87.44–99.42)
	Insect vector	1912.5 (1502.6–2390.9)	0.06 (0.03–0.11)	0
	Robot	1500.0 (1189.5–1860.3)	0.04 (0.01–0.07)	0

#### Dispersal with wind

In this test, a stable wind was generated by an axial fan that was installed at one end of the PMMA tube. The average wind speed was 2.5 m/s. The wind speed of 2.5 m/s was used based on pilot observations and previous research which has revealed that the mites disperse intentionally, not accidentally, in these conditions (Laska et al. 2019). Moreover, this wind speed corresponds to the mean wind speed during the summer in Poland (2.62 m/s) when cereal-feeding arthropods undertake dispersal before harvest. The information on the mean wind speed is based on the data collected at 61 meteorological stations in Poland, sourced from the Polish Institute of Meteorology and Water Management—National Research Institute.

**Fig. 1:**
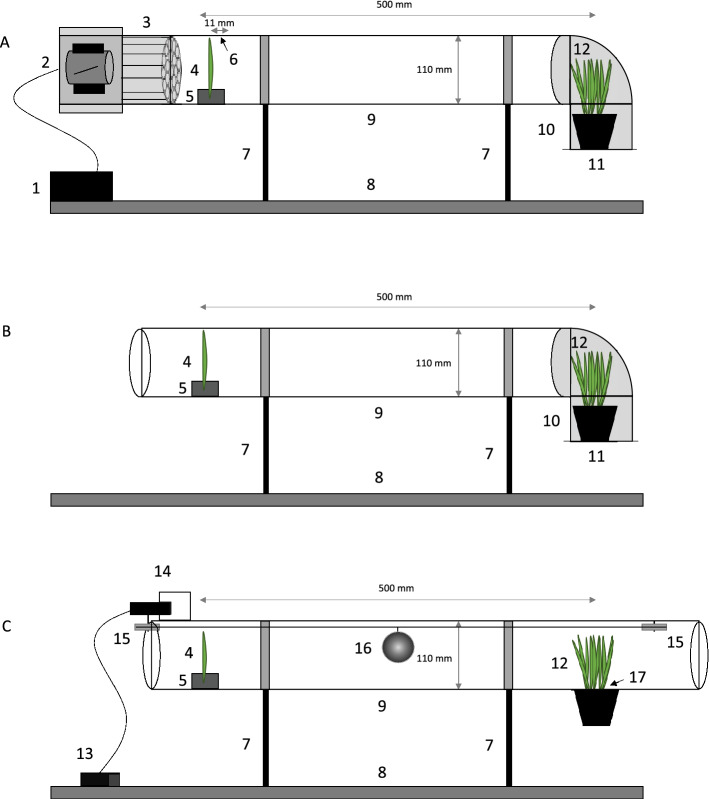
Scheme of testing dispersal with wind (**A**), insect vector (**B**) and robot (**C**). (1) power regulator controlling wind speed, (2) axial fan, (3) flow straightener consisting of small aluminium tubes that form a honeycomb-like structure in the cross-section, (4) source wheat shoot, (5) clips to keep source shoot upright, (6) hole for wind speed measurement by anemometer (hole was closed in the insect vector variant), (7) brackets with clamps, (8) platform, (9) transparent PMMA tube, (10) elbow connector, (11) metal grille, (12) the target wheat plants growing in pots, (13) control panel, (14) engine, (15) pulleys, (16) an artificial vector (spherical element of 4 cm diameter covered with wool to imitate a mammalian vector), (17) hole in the tunnel to install target plants

The source shoots were installed at the fan end of the wind tunnel. The mean source population sizes were 1290 for WCM and 1585 for CRM (Table 1). At the other end of the tunnel, target plants were placed on a metal grille in an elbow connector and were protected within a polyamide funnel to prevent contamination during the experiments (Fig. 1A) (for details see Kuczyński et al. 2020; Majer et al. 2021a). The experiment was replicated 10× for each species, and trials for each species were run separately, but under similar room conditions (Majer et al. 2021b).

#### Dispersal with an insect vector

*Anaphothrips obscurus* was used as an insect vector for mite dispersal. The thrips originated from quack grass collected in Huby Moraskie, Poland (52°28′04.3″ N, 16°55′36.2″ E), and were identified based on their morphology, according to Mirab-balou et al. (2013). For this purpose, thrips specimens were mounted on microscopic slides and examined under a compound microscope (Olympus BX41). Stock colonies of thrips were established by inspecting shoots using a stereomicroscope (Olympus SZ40), and transferring the insects onto potted wheat plants by using a small paintbrush. Thrips stock colonies were kept on wheat in rearing cages consisting of metal frames wrapped with nylon bags (to protect against contamination), at room temperature (22–24 °C), for the time of conducting experiments (ca. 6 months). *Anaphothrips obscurus* was used as a potential vector in eriophyoid mite dispersal because it is a host generalist and the most abundant thrips species on grasses, including cereals, in Poland. Moreover, *A. obscurus* inhabits and explores the same plant microhabitats as WCM and CRM (leaf sheaths and bases, leaf furrows, inner parts of the leaf), which increases the probability of contact between mites and thrips. Preliminary observations were performed, based on the investigation of up to 4000 shoots of wheat and triticale collected in cereal fields across Poland (ca. 312,000 km^2^) during three seasons, from June to August, 2012–2014. These observations indicated that both mite species and thrips co-occurred on wheat leaves and spikes, and WCM and CRM individuals were found attached to the legs, abdomens and wings of the thrips. Laboratory observations of thrips (*A. obscurus*) and mites (WCM or CRM) confirmed the field observations. Individual mites of both species were separately placed on thrips’ abdomens and the thrips and mite behaviour was observed. The thrips did not indicate ‘cleaning’ behaviours, but the mites also did not climb onto the vectors intentionally. A similar approach has been taken with aphids, but aphids performed ‘cleaning’ behaviours that removed mites from their bodies. Slykhuis (1955) also found WCM attached to thrips sampled in the field.

To establish thrips into mite source colonies, wheat shoots infested with mites were transplanted into small pots (tube-shaped) filled with soil (15 mm diameter) and covered with silicone tubes (to prevent contamination). Next, 24 h before the dispersal experiment was started, 10 thrips individuals (five winged and five wingless) from the laboratory stock colony were placed on each wheat shoot by using a small paintbrush. Winged and wingless thrips were used because in our pilot survey we observed mites attached to both forms. Both forms might act as potential vectors, as winged thrips are better dispersers, but wingless stages are more abundant. Mean mite populations on source shoots were 1079 for WCM and 1912.5 for CRM (Table 1). Before initiating the study in the tunnel, mites and thrips were incubated in laboratory conditions to allow acclimatisation and contact between mites and insects. After 24 h, wheat source shoots were examined under a stereomicroscope to confirm that thrips were still present with no escapes. Subsequently, these source shoots were installed into the tunnel, allowing thrips to move between the source shoot and target plants (Fig. 1B). Target plants were placed on a metal grille in an elbow connector and were protected within a polyamide funnel to prevent contamination during the experiments. No wind was generated. After the end of the experiment, target plants were inspected for thrips presence. In all repetitions, thrips (both winged and wingless) were found on the target plants (suggesting the dispersal of thrips). The experiment was replicated 10×  for WCM and 8× for CRM, and each species was tested separately, in similar room conditions.

#### Dispersal with a robot vector

In this test, we used the device described in Kuczyński et al. (2020), with some modifications. Lego Mindstorms NXT v.2.0 elements were used to construct a dispersal agent that could imitate a vector larger that an insect, such as a mammal. At the top of the tunnel, we placed two pulleys connected by a flexible string and attached an engine to one of the pulleys. On the string, a spherical element (4 cm diameter) was hung, covered with natural sheep felted wool material (20 × 20 cm, sourced from the common fabric shop). As it moved during the experiment, the element was allowed to contact both the source shoot and target plants. Mean mite populations of source shoots were 1595 for WCM and 1500 for CRM (Table 1). Shoots were installed as in previous treatments, whereas target plants were installed through a hole cut in the tube (Fig. 1C). The robotic engine was programmed to imitate the movement scheme of simplified natural activity of vertebrates that are present in the field and hypothetically could act as vectors of eriophyoid mites. The artificial vector moved through the middle of the tube toward an infested source plant and made contact with the source plant for 3 s and then moved in the opposite direction, toward the target plant. The vector contacted the target plants for 3 s. After that time, it returned to the centre of the tube and stopped for 30 min before repeating the same cycle. The robot was programmed to perform 24 cycles per 12 h of vectoring, followed by 12 h stationary in the centre of the tube. As the treatment exposure lasted 48 h, the whole programme (12 h vectoring and 12 h stationary) was repeated on the second day. The experiment (the whole 48 h programme) was replicated 10×  for WCM and 11×  for CRM, with each replicate and each species tested separately in similar room conditions.

#### Incubation of target plants

After 48 h of dispersal, all target plants were covered with nylon bags to avoid contamination, transferred into a growth chamber and incubated for 14 days at ambient conditions (22–24 °C, L12:D12 photoperiod, 45% RH). During that time, mites that successfully dispersed toward target plants were allowed to reproduce. After 14 days, the mites on each target plant (i.e., the colonists, *C*) were counted. The 2-week incubation period was used to estimate the number of individuals that successfully established on the target plants (founders) (as described in Kuczyński et al. 2020).

### Statistical analysis

By knowing the number of colonists (*C*) after 2 weeks and the population growth rate (*r*) at a specific rearing temperature (Kuczyński et al. 2016), we estimated the number of individuals that successfully settled and established the population on the target plant (number of founders, *F*). Thus, *F* was an unobservable (latent) variable. Dispersal success (*d*) was calculated according to the formula *d* = *F*/*N*, i.e., the proportion of founders to the number of individuals in the source population.

To calculate the model parameters, we used a Bayesian hierarchical modelling approach, as described in Kuczyński et al. (2020) and modified in Majer et al. (2021a). Dispersal success (*d*) of WCM and CRM using different dispersal modes was estimated independently. Statistical analyses were performed using JAGS Gibbs-sampling environment (Plummer 2003) and R v.3.6 (R Foundation for Statistical Computing, Vienna, Austria 2019). Both environments were integrated via the jags UI library (Kellner 2018). Vague normal density priors were used for the logit-scale parameters for expected probability of dispersal success, and half-Cauchy density priors were used for the logit-scale standard deviations of random intercepts. For population growth rates, informative priors were used, which were adopted from Kuczyński et al. (2016). Gibbs sampling was performed with three independent chains for 1.2 × 10^6^ iterations each. The first 2 × 10^5^ iterations of each chain were discarded as burn-in, and only samples from every 100th iteration were stored.

## Results

**Fig. 2:**
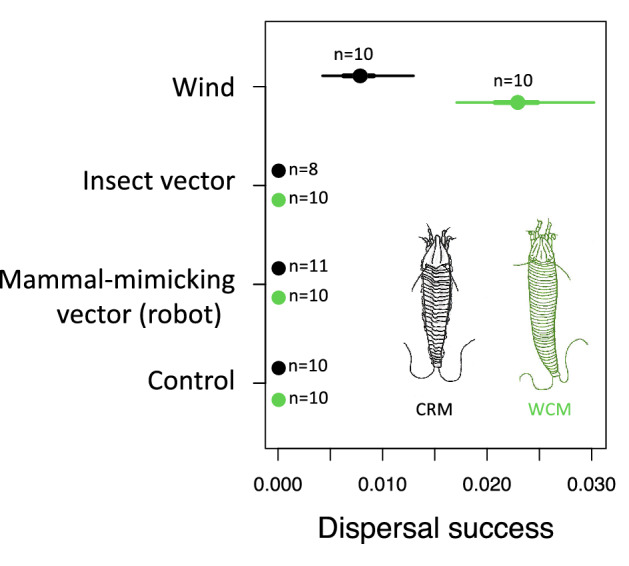
Dispersal success of *Aceria tosichella*, WCM (green) and *Abacarus hystrix*, CRM (black) using three dispersal agents, namely wind, insect vector, and mammal-mimicking vector (robot). No dispersal agent was used for the control. The dots denote means, thick error bar lines are 50%, and thin error bar lines are 95% credible intervals for the estimate. No dispersal events were found for both control and phoretic treatments, thus there are no error bars around their means. The number of replicates for each test is indicated by ‘n’

In both WCM and CRM, dispersal was successful only with wind. No mite dispersal was detected with the insect vector or the robot, or in the control (Table 1, Fig. 2). The success of WCM aerial dispersal [*d* = 0.023, 95% credible interval (CI) 0.017–0.030] was significantly higher than that of CRM (*d* = 0.008, 95% CI 0.005–0.014) (Fig. 2).

During dispersal sessions, the source shoots have wilted and dried out gradually, and were completely dry after the sessions. Most of the resident mite individuals on the source shoots were dead after each of the dispersal session. Single live individuals (both mobile and quiescent) were found on source shoots in all tests except the aerial dispersal of CRM (in that treatment, all residents died—no live mites were found on the source shoots) (Table 1).

## Discussion

The knowledge of dispersal strategies of pest species is of special importance for improving pest control strategies. Here, we investigated the dispersal success of two economically important eriophyoid mites (WCM MT-1 and CRM complex 2) that are wheat pests, by comparing different dispersal modes: aerial dispersal and phoretic dispersal with large and small vectors. We found wind to be the only successful factor in passive dispersal of both species, as we did not detect phoretic dispersal with the tested vectors.

The two eriophyoid species studied significantly differed in their aerial dispersal success; WCM dispersed more effectively than CRM. Majer et al. (2021a) also demonstrated that aerially transferred WCM have a higher colonisation ability than CRM. WCM also showed increased behavioural activity via wind, whereas the activity of CRM decreases in such conditions (Kiedrowicz et al. 2017). Thus, the results of this study are consistent with previous investigations. However, it may be expected that CRM would have lower mortality during aerial dispersal than WCM, because CRM are known to produce wax filaments and this is hypothesised to regulate water loss and maximise the drag during aerial dispersal. Frost (1997) has experimentally shown that the wax filaments produced by CRM sensu lato increase the survival of individuals at a low relative humidity and increase the non-cuticular surface area. In our study, however, all CRM resident individuals died on the source shoots after 2 days of wind exposure as no live residents were observed on the source shoots. In contrast, some WCM individuals remained alive on the source shoots, although WCM does not produce wax filaments. Wosula et al. (2015) obtained similar results when they compared WCM off-host survival with the CRM sensu lato survival tested by Frost (1997) and found that CRM survival is not significantly greater than that for WCM. However, WCM sensu lato is less vagrant than CRM sensu lato (Sabelis and Bruin 1996), as it usually hides within the leaf sheaths; therefore, it is less exposed to unfavourable abiotic conditions on the host plant than CRM. It is possible that WCM residents survived better on the dried source shoots than CRM, as WCM regularly hide in curled leaves or leaf sheaths (Nault and Styer 1969). Moreover, it has been suggested that factors such as drying of the host plants, but also increased temperature and light, may increase the dispersal of WCM (Nault and Styer 1969). The differential effects of these abiotic factors for CRM and WCM remain unknown. Due to the fact that different biotic and abiotic conditions might influence dispersal potential of both species, further studies that include testing such factors are necessary.

Transfer with wind currents has been regarded as a risky mode of dispersal (Sabelis and Bruin 1996), as organisms cannot control the direction of movement, and there is a very low probability of landing on a suitable patch. Thus, aerial dispersal is extremely adventurous for highly specialised animals, and would be more appropriate for use by generalists (Sabelis and Bruin 1996; Bonte et al. 2003). WCM sensu lato and CRM sensu lato have long been regarded as host generalists that can inhabit several dozen grass species (Amrine and Stasny 1994; Navia et al. 2013), and thus, they have been considered examples that support the Sabelis and Bruin (1996) hypothesis. However, recent studies revealed that WCM sensu lato and CRM sensu lato are, in fact, species complexes consisting of several cryptic lineages, and particular lineages of each species inhabit a smaller number of host species than previously thought. Specifically, WCM MT-1 and CRM complex 2 to date have been found on nine and four grass species, respectively (Laska et al. 2018; Skoracka et al. 2018a). WCM MT-1 has also survived on plants belonging to the Amaryllidaceae family (Skoracka et al. 2014a). The high aerial dispersal ability in lineages of WCM and CRM may be associated with their capability to feed on different hosts, but it likely also results from other ecological factors, such as the annual dynamics of agroecosystems especially the seasonal variation in cereal host availability.

At a local scale, cereals provide a relatively stable environment that is highly homogeneous in terms of quality and the spatial distribution of hosts, and they provide a high-level resource for phytophagous consumers for a part of the year (Vialatte et al. 2005; Lombaert et al. 2006). The high availability of appropriate habitats could be a factor increasing successful aerial dispersal by decreasing the risk of landing in an unfavourable place, and this should accelerate the evolution of aerial dispersal (Sabelis and Bruin 1996; Bowler and Benton 2005). Moreover, some have suggested that aerial dispersal of WCM sensu lato might be directed, as mite movement depends on the wind direction in the field (Umina et al. 2016). This would also reduce the chances of landing in an unfavourable place. However, cereal resources are not available between harvest and planting of the subsequent crop (Wegulo et al. 2008; Gillespie et al. 1997). As harvest approaches, mites must disperse and colonise alternative hosts rapidly, and wind is the most ubiquitous agent for passive dispersal in the field (Navia et al. 2013; Wosula et al. 2015; McMechan and Hein 2017). The shift from cereals to alternative hosts enabled by the wind is risky because aerial dispersal is characterised by a low degree of control of the direction and distance of travel, and high mortality (Bonte et al. 2012). Eriophyoid mites may compensate for these costs of aerial dispersal by their short developmental time, arrhenotoky, high intrinsic population growth rate, and high colonisation abilities (Navia et al. 2013). It has been shown that WCM sensu lato may reach very high population densities just prior to harvest (McMechan and Hein 2017), and mite dispersal during this time would certainly be impacted by the plant growth stage and environmental conditions (Umina et al. 2016). Agroecosystems are, therefore, habitats that favour phytophagous arthropods with a high rate of population increase and with dispersal strategies adapted to both the optimal exploitation of locally abundant resources and the rapid colonisation of fluctuating environments (Lombaert et al. 2006). Thus, we can conclude that passive aerial dispersal is the most advantageous mode of dispersal for WCM and CRM.

Phoretic dispersal has also been discussed to play some role in eriophyoid mites spreading (Sabelis and Bruin 1996; Michalska et al. 2010). On the one hand, eriophyoid mites have been observed attached to the bodies of many vectors, both small (e.g., spiders and insects) and large (e.g., humans), suggesting the possibility of phoresy (Michalska et al. 2010). For example, the coconut mite, *Aceria guerreronis* Keifer was found on bees, ants, and other insects, but also on bats (Julia and Mariau 1979; Griffith 1984; Moore and Alexander 1987; Schliesske 1990; Sumangala and Haq 2005; Galvão et al. 2012). The grape rust mite, *Calepitrimerus vitis* (Nalepa), was found attached on human hands and transported between plants by humans as vectors (Duffner et al. 2001). On the other hand, most of the conclusions on phoretic dispersal in eriophyoid mites were based on reports about mites attached to the bodies of potential vectors, without restrictive testing of the possibility to disperse from site to site (Michalska et al. 2010). Moreover, Lindquist and Oldfield (1996) argued that eriophyoid mites have no clear morphological adaptations allowing effective phoretic dispersal. Kiedrowicz et al. (2017) examined the behaviour of WCM and CRM in the presence of a potential insect vector and did not detect any behavioural adaptations to phoresy. The lack of adaptations to phoresy might suggest that this is rather an accidental and inefficient dispersal mode. However, Liu et al. (2016) showed that an eriophyoid mite species, *Aceria pallida* Keifer, effectively disperses attached to the psyllid *Bactericera gobica* (Loginova) and, moreover, uses this insect as a winter hibernation site, successfully escaping unfavourable winter conditions and returning to its host plant early in the spring. This obligatory interaction of the eriophyoid mite and insect indicates that phoresy can evolve in eriophyoid mites. However, the existence of phoresy in most eriophyoid mite species requires further investigation.

In this study we tested whether WCM and CRM might disperse with small or large vectors, as this phenomenon has been scarcely investigated in grass-feeding mites. The possibility of WCM sensu lato to disperse on insects was earlier investigated in a greenhouse experiment, which showed that mites rarely disperse with aphids (Gibson and Painter 1957). In our study, we used thrips as small vectors in tests of phoretic dispersal of WCM and CRM. Thrips and mites co-occur in sympatry, so there is a high probability of direct contact between them (Michalska et al. 2010; personal observations). We also used a robot to imitate a large vector (such as a mammal). It has been found that some eriophyoid mites could be transported by human activities (Barke et al. 1972; Schliesske 1977; Tanaka and Shibao 2003; Duffner et al. 2001; Gabi and Mészáros 2003), thus it would be also possible that cereal-feeding mites disperse with vertebrates associated with cereal fields, such as roe deer, foxes, hares and rodents. However, our results did not confirm efficient phoresy in WCM nor CRM. Interestingly, in other studies that used a robot to test phoretic dispersal in WCM (Kuczyński et al. 2020), there was some evidence for dispersal with a robot, but it was an extremely rare event. Our results confirm that in WCM and CRM successful settlement after transportation with vectors is unlikely.

Studying passive dispersal is logistically challenging, especially in minute invertebrates such as eriophyoid mites, because tracking their movement is difficult. Thus, providing experimental data on their dispersal strategies will help to understand the mechanisms of their spread, and could be useful to predict their range expansion and distribution in the field. It has been shown in many other taxa that the basic knowledge of their biology and ecology, including mechanism of their dispersal may help to predict and prevent future invasions (Drake and Lodge 2006; Liebhold and Tobin 2008; Catford et al. 2011; Frost et al. 2019; Dominguez Almela et al. 2020). Information on dispersal modes and strategies could be incorporated into models of pest spreading and outbreaks, and may be helpful in monitoring and predicting future invasions (Jeger 1999). The enhanced dispersal potential of WCM shown in this study may also impact range expansion, increasing the risk of its invasion (Skoracka et al. 2014b).

## Data Availability

The data that support the findings of this study are openly available in the Zenodo repository under: http://doi.org/10.5281/zenodo.4719293.

## Code availability

Custom code used in this study is openly available in the Zenodo repository under: https://doi.org/10.5281/zenodo.3964151.
